# Cell-Free DNA Sequencing Reveals Gene Variants in DNA Damage Repair Genes Associated with Prognosis of Prostate Cancer Patients

**DOI:** 10.3390/cells11223618

**Published:** 2022-11-15

**Authors:** Verena Lieb, Amer Abdulrahman, Katrin Weigelt, Siegfried Hauch, Michael Gombert, Juan Guzman, Laura Bellut, Peter J. Goebell, Robert Stöhr, Arndt Hartmann, Bernd Wullich, Helge Taubert, Sven Wach

**Affiliations:** 1 Department of Urology and Pediatric Urology, Universitätsklinikum Erlangen, Friedrich-Alexander-Universität Erlangen-Nürnberg, 91054 Erlangen, Germany; 2 Comprehensive Cancer Center Erlangen-EMN (CCC ER-EMN), 91054 Erlangen, Germany; 3 QIAGEN GmbH, 40724 Hilden, Germany; 4 Institute of Pathology, University Hospital Erlangen, FAU Erlangen-Nürnberg, 91054 Erlangen, Germany

**Keywords:** prostate cancer, cfDNA, sequence variants, DDR genes, ATM, NBN, prognosis

## Abstract

In the present study, we further analyzed the data obtained in our previous study, where we investigated the cell-free DNA (cfDNA) of 34 progressive prostate cancer patients via targeted sequencing. Here, we studied the occurrence and prognostic impact of sequence variants according to their clinical pathological significance (CPS) or their functional impact (FI) in 23 DNA damage repair (DDR) genes with a focus on the ATM serine/threonine kinase gene (ATM). All patients had at least one DDR gene with a CPS or FI variant. Kaplan-Meier analysis indicated that the group with a higher number of CPS variants in DDR genes had a shorter time to treatment change (TTC) compared to the group with a lower number of CPS variants (*p* = 0.038). Analysis of each DDR gene revealed that CPS variants in the ATM gene and FI variants in the nibrin (NBN) gene showed a shorter TTC (*p* = 0.034 and *p* = 0.042). In addition, patients with CPS variants in the ATM gene had shorter overall survival (OS; *p* = 0.022) and disease-specific survival (DSS; *p* = 0.010) than patients without these variants. Interestingly, patients with CPS variants in seven DDR genes possessed a better OS (*p* = 0.008) and DSS (*p* = 0.009), and patients with FI variants in four DDR genes showed a better OS (*p* = 0.007) and DSS (*p* = 0.008). Together, these findings demonstrated that the analysis of cfDNA for gene variants in DDR genes provides prognostic information that may be helpful for future temporal and targeted treatment decisions for advanced PCa patients.

## 1. Introduction

Prostate cancer (PCa), with approximately 1.4 million men diagnosed and approximately 375,000 men succumbing to PCa in 2020, remains a major cause of disease and mortality among men worldwide [1]. Genetic studies of PCa have revealed DNA alterations that dysregulate genes involved in androgen signaling, the TP53 pathway, cell cycle regulation, the PI3K pathway, the WNT pathway, chromatin modification, DNA damage repair (DDR) and other pathways [2,3,4,5]. Functionally intact DDR pathways provide an efficient anticancer barrier [6], but genome instability and mutations enable characteristics of tumor development, especially defects in components of the DNA maintenance machinery supporting this development [7]. The inactivation of certain components of these pathways is a prerequisite for malignant transformation (reviewed in [8]).

Genetic studies are mainly based on biopsy or prostatectomy specimens. However, an easily accessible source for genetic information is cell-free DNA (cfDNA), which is ubiquitous in body fluids, such as blood (serum or plasma), urine, cerebrospinal fluid, saliva, sperm and others. cfDNA is mostly derived from apoptotic or necrotic cells originating from hematopoietic cells, stromal cells and endothelial cells, but in cancer patients, it is also derived from primary, relapsed or metastatic tumor cells [9]. In addition, the release of tumor cfDNA by the active secretion of extracellular vesicles has been suggested [10]. Genetic and epigenetic alterations in cfDNA provide clinically useful tumor markers for early diagnostics, monitoring of tumor progression (including the development of resistance mechanisms in real time), and evaluation of therapy response and guidance for therapy choice [11,12]. In PCa, the quantity of cfDNA has diagnostic potential as it has been shown that it is, on average, higher in PCa patients than in BPH patients or control probands [13,14,15,16,17]. Moreover, an association between high cfDNA levels, including circulating tumor DNA (ctDNA), and poor prognosis of PCa patients has been reported and correlated with the overall tumor burden in castration-resistant PCa patients [16,18,19,20]. In these patients, several genomic alterations have been detected in the cfDNA/ctDNA for the androgen receptor, which are associated with the outcome of anti-androgen therapies, as well as for DNA damage repair genes associated with the response to PARP inhibitors [21,22,23,24,25]. Genomic analysis of ctDNA from patients with mCRPC recapitulates the genomic landscape detected in tissue biopsies, i.e., a high concordance is observed but more acquired resistance alterations of the BRCA1/2 genes are detected in ctDNA than in tissue biopsies [25]. Interestingly, mutations in DDR genes do not always relate to a comparable response to olaparib, a PARP inhibitor. A differential response to olaparib treatment among men with metastatic castration-resistant prostate cancer harboring BRCA1/2 versus ATM mutations has been observed, in which patients with BRCA1/2 mutations respond to olaparib treatment but not those with ATM mutations [26]. Although none of the patients received olaparib in our study, we are interested in whether gene variants in the ATM gene are associated with the prognosis of PCa patients. ATM serine/threonine kinase (ATM; also known as ataxia-telangiectasia mutated gene) is a 350 kDa protein that contains 3056 amino acids, and it belongs to the phosphatidylinositol 3-kinase (PI3K) family [27,28]. ATM is a signaling kinase that is activated by DNA double-strand breaks, and it plays a major role in DDR, i.e., double-strand DNA damage repair, both in nonhomologous end-joining (NHEJ) in the G1 phase of the cell cycle and in homologous recombination (HR) in the S and G2 phases of the cell cycle [29]. ATM inactivation is a crucial step in promoting androgen-induced genomic instability and prostate carcinogenesis [30]. ATM gene variants contribute to PCa susceptibility and progression, particularly aggressive PCa [4,31,32]. Among the mutated DDR genes, the ATM gene is mutated in advanced PCa with an approximate mutation rate of 7.3%, which is only exceeded by BRCA2 mutations with a mutation rate of 13.3% [33].

In the present study, we investigated the occurrence and prognostic impact of DDR gene variants with a special focus on ATM gene variants detected in cfDNA of PCa patients.

## 2. Materials and Methods

### Patients and Tumor Material

The dataset underlying this analysis has been described in detail [34]. Briefly, targeted NGS sequencing was conducted using 39 samples of cfDNA originating from 34 PCa patients. An overview of the clinicopathological data, treatment and study data of the patients is provided in Table S1. There was a total of 99 genes, including 93 genes in the breast cancer panel (DHS-001Z-96; Qiagen) and 6 additional PCa-relevant genes (TMPRSS2, ERG, ERCC1, ERCC3, FOXA1 and SPOP). The identified variants were further analyzed for their clinical or functional impact using the QCI translational application (QIAGEN Clinical Insight Interpret 8.0.20210827). This QCI translational application allows classification of gene variants by their clinic pathological significance (pathogenic, likely pathogenic, benign, likely benign variants or variants with uncertain significance) and by their functional impact (deletion or gain of function, normal function).

The times for prognosis analysis were for overall survival (OS) from tumor diagnosis to death of any reason or to the last follow up, for disease-specific survival (DSS) from tumor diagnosis to death reasoned by the tumor or to last follow up, and for time to treatment change (TTC) from time of blood sampling until treatment change. The associations of sequencing results with OS, DSS and TTC were determined by univariate analyses (Kaplan–Meier analysis with log-rank test and Cox’s regression hazard models). A *p* value less than 0.05 was considered statistically significant. Statistical analyses were performed using the SPSS 21.0 software package (SPSS Inc., Chicago, IL, USA).

## 3. Results

The cfDNA of 34 PCa patients (39 samples) was evaluated for gene variants in 99 genes by NGS as described previously [34] and here in Table S2. We investigated in a continued analysis, gene variants in DNA damage repair genes and their association with prognosis, i.e., OS, DSS or TTC. Gene variants were evaluated with respect to their clinical pathological significance (CPS), i.e., if variants have a described pathogenic or likely pathogenic effect or their functional impact (FI) as described by a predicted loss or gain of function irrespective of a potential pathological impact. The ATM gene within the DDR genes was focused on due to the not comprehensively characterized role of mutations in the ATM gene and their association with the prognosis of PCa.

### 3.1. Molecular Characteristics of Tumors

All analyzed samples exhibited at least one DDR gene containing a variant with CPS or FI. Out of the 23 DDR genes included in the gene panel, patients showed CPS variants in 19 genes and FI variants in 22 genes as shown in Table S3. For the ATM gene, 7 CPS variants were detected in 6 patients, and 26 FI variants were identified in 20 patients. All ATM CPS variants also showed an FI.

### 3.2. Association of DDR Gene Variants with Prognosis

**Figure 1:**
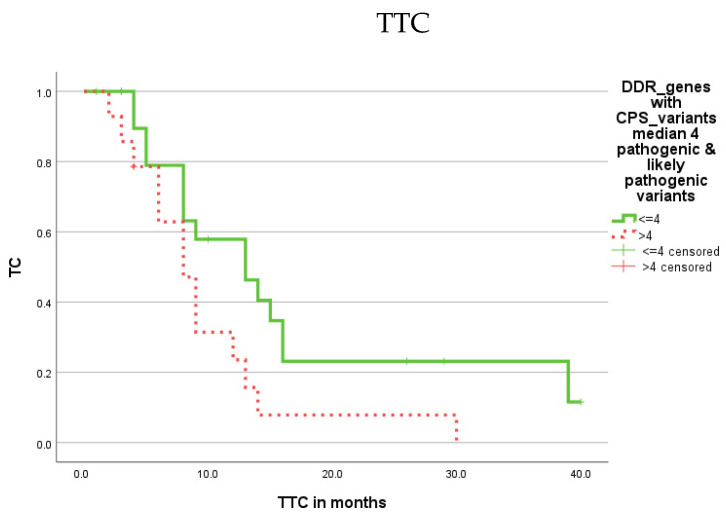
Kaplan–Meier analysis showing the association of CPS variants in DDR genes with prognosis (TTC). Patients with CPS variants in a higher number of DDR genes (>4; red dotted) showed a shorter TTC (*p* = 0.038) compared to patients with a lower number of affected DDR genes (≤4; green solid).

We first investigated whether the number of gene variants either with CPS or FI was associated with prognosis, i.e., OS, DSS or TTC. Patients were stratified according to their number (median) of DDR genes affected by variants with >4 vs. ≤4 vs. for CPS and >7 vs. ≤7 for FI. There was no difference based on the number of variants within the CPS or FI groups regarding OS or DSS. However, a higher number of affected DDR genes with variants in the CPS classification was associated with a shorter TTC compared to the group with a lower number of affected DDR genes with CPS variants according to the Kaplan-Meier analysis (9.7 vs. 16.9 months; *p* = 0.038; Figure 1; Table 1).

**Figure 2:**
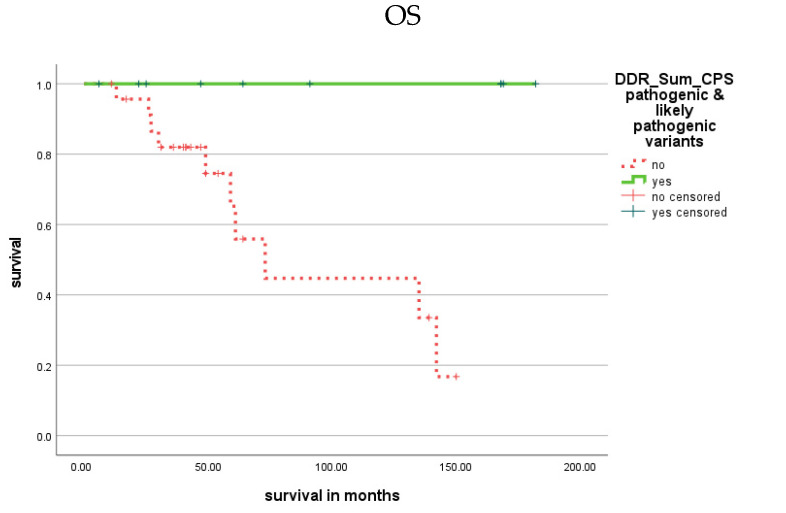

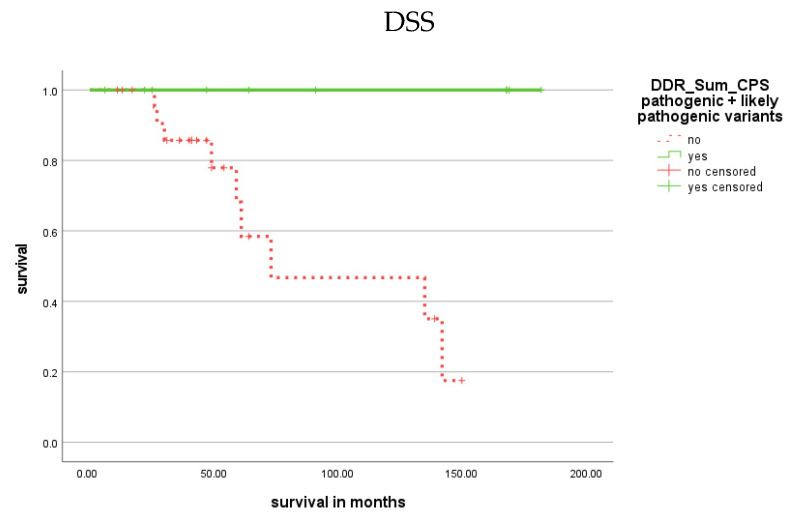
Kaplan–Meier analysis showing the association of CPS variants in some DDR genes with prognosis (OS and DSS). Patients with CPS variants in some DDR genes (MSH6, ERCC1, ERCC3, ERCC4, PMS1, NBN and FANCC; green solid) had a better OS (*p* = 0.008) and DSS (*p* = 0.009) than patients with CPS variants in other DDR genes (red dotted).

**Figure 3:**
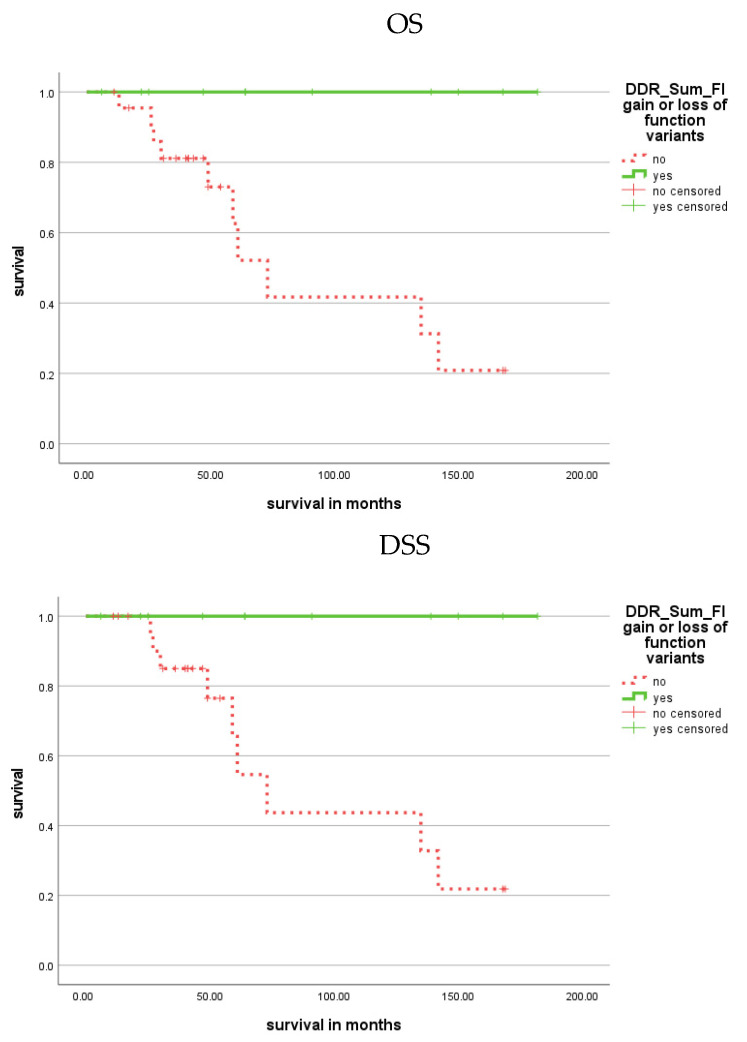
Kaplan–Meier analysis showing the association of FI variants in some DDR genes with prognosis (OS and DSS). Patients with FI variants in some DDR genes (MLH1, ERCC1, ERCC4 and FANCC) had a better OS (*p* = 0.007; green solid) and DSS (*p* = 0.008; green solid) than patients with FI variants in other DDR genes (red dotted).

We next analyzed whether any of the single DDR genes were associated with prognosis. Except for variants of the ATM gene or the NBN gene (see below), no association with prognosis was found for variants in any other DDR gene. However, we observed that variants in some DDR genes were associated with a favorable OS or DSS. Therefore, we evaluated patients with these gene variants, i.e., CPS variants in seven genes (MSH6, ERCC1, ERCC3, ERCC4, PMS1, NBN and FANCC) and FI variants in four genes (MLH1, ERCC1, ERCC4 and FANCC; Table S3). Kaplan-Meier analysis indicated that patients with these CPS variants had a better OS (*p* = 0.008) and DSS (*p* = 0.009; Figure 2; Table 1) and that patients with these FI variants showed a better OS (*p* = 0.007) and DSS (*p* = 0.008; Figure 3; Table 1). However, such an association was not observed for these gene variants and TTC.

### 3.3. Association of ATM Gene Variants with Prognosis

**Figure 4:**
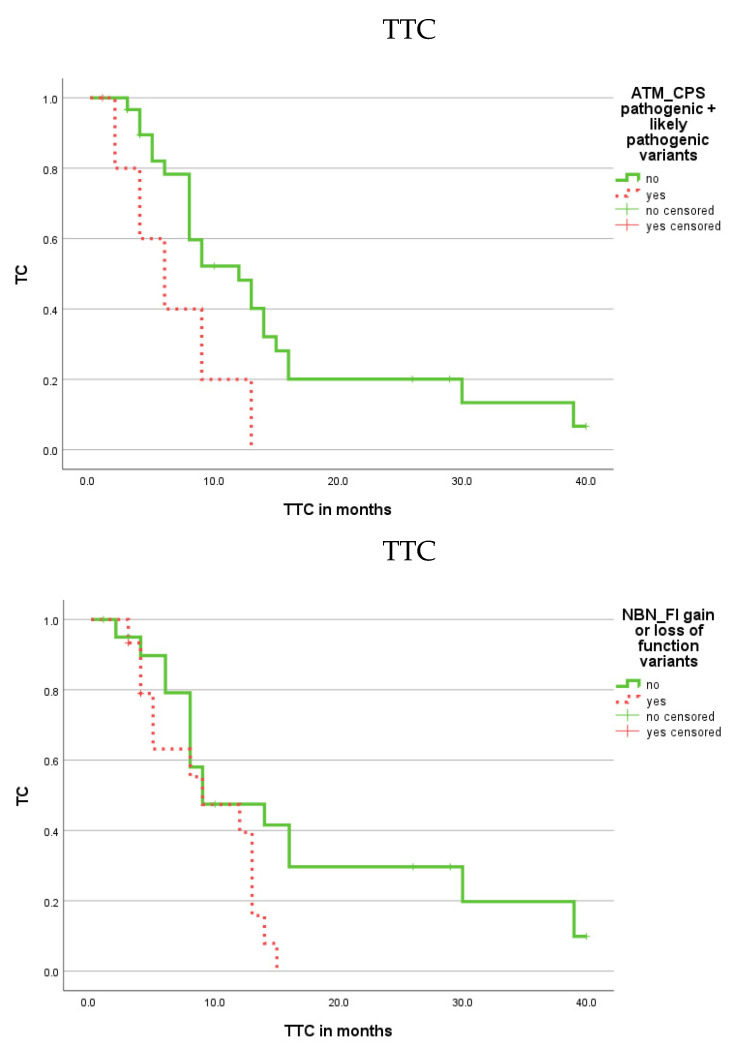
Kaplan–Meier analysis showing the association of CPS variants in the ATM gene and FI variants in the NBN gene with prognosis (TTC). Patients with CPS variants in the ATM gene or with FI variants in the NBN gene had a shorter TTC (*p* = 0.034 and *p* = 0.042; red dotted).

Kaplan–Meier analysis demonstrated that patients with CPS variants in the ATM gene showed a shorter TTC (*p* = 0.034; Figure 4; Table 1), but no association between FI variants and TTC was found.

**Figure 5:**
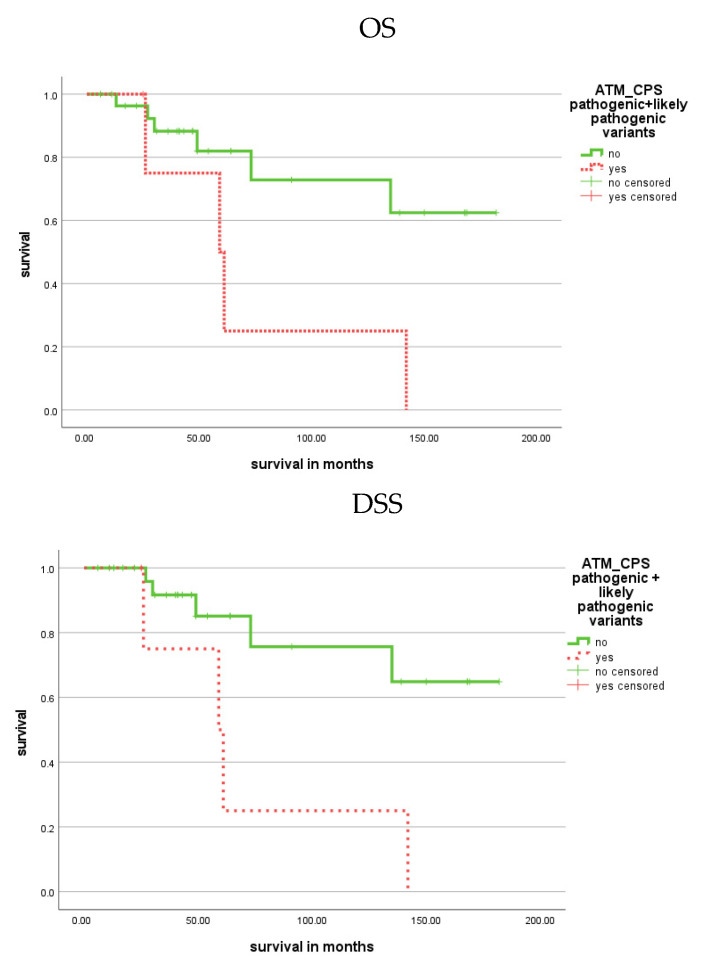
Kaplan–Meier analysis showing the association of CPS variants in the ATM gene with prognosis (OS and DSS). Patients with CPS gene variants in the ATM gene had a worse OS and DSS than patients without these gene variants (*p* = 0.022 and *p* = 0.010; red dotted).

In addition, patients with CPS variants in the ATM gene had a shorter OS (*p* = 0.022) and DSS (*p* = 0.010; Figure 5; Table 1) than patients without these variants (Table 1). In univariate Cox regression analysis, the occurrence of CPS variants was associated with a 3.96-fold increased risk of death (*p* = 0.034) and a 4.82-fold increased risk for tumor-related death (*p* = 0.020) in PCa patients compared to patients without these variants (Table 2).

### 3.4. Association of NBN Gene Variants with Prognosis

Patients with FI variants in the nibrin (NBN) gene had a shorter TTC (*p* = 0.042; Figure 4; Table 1). However, there was no association for CPS variants in the NBN gene with TTC as well as no association of CPS/FI variants in the NBN gene with OS or DSS.

## 4. Discussion

DDR plays an important role in PCa biology and in the development of resistance mechanisms [4,5,33,35]. In general, inherited mutations in DNA repair genes, such as BRCA2, are associated with increased risks of lethal prostate cancer [36]. In addition, it has been recently reported that a substantial proportion of the primary tumors of patients undergoing radical prostatectomy harbor mutations in DNA damage repair genes, which is associated with shorter progression-free survival [37]. However, the prognosis of men with PCa with mutations in DNA damage repair (DDR) genes undergoing different treatment schemes is still unclear [38]. It is important to note that DDR gene status is concordant between archival primary tissue taken at cancer diagnosis and serial ctDNA-positive samples collected in the mCRPC setting [39]. Furthermore, 90% of somatic mutations present in matched metastatic tissue are also detected in ctDNA [19].

Recently, the analysis of ctDNA for alterations in homologous recombination (HR) repair genes in PCa has been reviewed [40]. However, the DDR comprises more repair genes and pathways. In double-strand DNA repair, in addition to HR genes, there are also genes involved in the nonhomologous end joining (NHEJ), alternative NHEJ and single-strand annealing (SSA) pathways. In addition, DDR genes are active in single-strand DNA repair (base excision repair), repair of bulky lesions (nucleotide excision repair), and nucleotide mismatches (mismatch repair) [33,35]. In the present study, we analyzed 23 DDR genes involved in different DDR pathways for gene variants and assessed their prognostic impact.

We detected gene variants with functional impact (FI) in 22 genes and with clinical pathogenic significance (CPS) in 19 genes (Table S3). After stratifying the patients at the median of affected DDR genes with CPS variants (>4 vs. ≤4), those with >4 affected DDR genes with CPS variants showed a shorter TTC compared to patients with fewer DDR genes with CPS variants. This finding agreed with our previous result that patients with a higher number of gene variants have a shorter TTC in general [34]. We evaluated the association of gene variants in each DDR gene with OS or DSS, and we found that patients with gene variants in some DDR genes showed a better OS or DSS than patients without such gene variants. We summarized the genes with these positive CPS variants (DDR_Sum_CPS: MSH6, ERCC1, ERCC3, ERCC4, PMS1, NBN and FANCC) or FI variants (DDR_Sum_FI: MLH1, ERCC1, ERCC4 and FANCC). As expected, patients with these CPS or FI variants had a significantly longer OS or DSS.

Similarly, Neviere et al. recently identified mutations in several DDR genes (ATM, BRCA1/2, FANC-C/-F/-G/-M, CHEK1/2, CDK12, MRE11A, PALB2 and BLM) in mCRPC patients; patients with DDR gene mutations showed somewhat better overall and progression-free survival than patients without these mutations, but the differences were not significant [41].

The positive prognostic impact on OS or DSS in the present study may be due to several factors. First, these gene variants pertain to different DNA repair pathways. While a functionally intact DDR system is considered a barrier against malignant progression [8], tumor cells commonly carry germline and/or somatic DDR gene mutations and/or develop DDR gene mutations in response to systemic treatment. However, as demonstrated by the efficacy of PARP inhibitors, especially in BRCA1/2-deficient tumors, a certain degree of DDR capacity must be maintained for genomic stability. If too many of the different DDR pathways are affected, this may render tumor cells susceptible to apoptosis. Second, defects in multiple DDR pathway genes may lead to enhanced presentation of tumor-related neoantigens and enhanced antitumor immune reactions. When we considered the number of gene variants in DDR genes at the patient level, we found no summation effect or association with OS or DSS, which argues against a simple association between DDR gene mutation frequency and OS or DSS. In addition, a high frequency of mutations, as occurs during chromothripsis in cancer, including PCa, in which tens to hundreds of genetic rearrangements can occur in a one-step cataclysmic process [42,43], argues against DDR gene mutation frequency. Rather, a hierarchical system may be possible with a different importance of DDR genes/gene variants for tumor cell survival and impact on patient’s prognosis or gene variants may affect most of the DDR pathways in a patient, which may be deleterious for tumor cell survival. However, these hypotheses have to be tested in larger studies in the future.

Both NBN and ATM gene variations were noticeable when studying single DDR gene variants for their association with prognosis. Patients with FI variants in the NBN gene had a shorter TTC. The NBN (Nibrin, synonymous: Nijmegen breakage syndrome 1/NBS1) protein is part of the HR system, repairing double-strand DNA breaks [44]. The NBN gene is a PCa susceptibility gene associated with aggressive disease [45], and it belongs to the network of DNA repair genes that are both induced by androgen and represent androgen receptor target genes [46]. NBN mutations have been reported in PCa tissue or as germline mutations of PCa patients [45,47] but not yet in cfDNA. Because NBN is active in HR in a complex (NBN/BRCA1/BRCA2/MRE11/RAD50/BLM/PALB2) [33], it would be of interest to determine whether PARP inhibitors have a therapeutic effect in patients with NBN gene variants/mutations.

In the present study, we showed for the first time that patients with CPS variants in the ATM gene detected in cfDNA had a shorter OS, DSS and TTC. In univariate Cox’s regression analyses, the presence of CPS variants was associated with a 3.96-fold increased risk for death and a 4.82-fold increased risk of tumor-related death. Mutational hot spots in the ATM gene have not yet been detected [27]. Interestingly, two missense gene variants, namely, 2572T/C (858 Phe>Leu) and 5557G/A1853 (Asp>Asn), have been identified in breast cancer cases [48] and in the present study, but they are not considered pathogenic/likely pathogenic. In a large study of 692 metastatic PCa patients, 11 ATM germline mutations (1.6%) were detected [36]. Only one mutation, a deletion starting in c.3764 (p. L1255*), was the same position as a missense mutation (c.3764T>G; p. L1255 W) in one patient in the present study, supporting the abovementioned finding that there are no hot spot mutations in the ATM gene [27]. Recently, Tolkach et al. showed that ATM mRNA is downregulated in the tumor tissue of CRPC patients or PCa patients treated with androgen deprivation therapy compared to primary PCa patients [49]. This is in line with our finding that ATM variants—mostly presenting loss of function variants—are associated with a poor prognosis.

Defects in BRCA2 and ATM are strongly associated with poor clinical outcomes, i.e., shorter progression-free survival [21]. However, a recent study has reported that mCRPC patients with somatic mutations in BRCA1/2 and ATM benefit from standard therapies and have a longer progression-free survival (long response to taxane therapy) than patients without these mutations [41]. Comparably, Kaur et al. showed that mCRPC patients with mutations in the BRCA1/2 or ATM gene who are treated with taxanes as the first-line therapy show a longer progression-free survival than patients without these alterations [50]. However, these researchers also demonstrated that ATM loss detected by immunohistochemistry is significantly associated with an increased risk of metastasis in univariate analysis but not after adjusting for Gleason grade [50].

By evaluating copy number alterations in ATM mutant PCa vs. HR-proficient, HR-deficient or BRCA2 mutant PCa, Ryan et al. reported that ATM-mutated PCa displays copy number alterations for the FGF19, FGF4, PTPN11, ALDH2, DAXX, BCL7A, CCND1, BMPR1A and MEF2B genes, suggesting that FGF- and PTPN11-related pathways are potentially targetable pathways in ATM mutant PCa [51].

Another possibility for PCa patients with ATM mutations is the therapeutic application of ATM inhibitors, such as AZD1390, AZD0156, M4076, Ku 60019 or XRD-0394, but these inhibitors are still in clinical phase I studies. However, the combination of the DNA-methylating drug, temozolomide, with the ATM inhibitor, KU60019, has been shown to result in an increased induction of apoptosis in glioblastoma cells in vitro [52]. Furthermore, Fischer et al. showed that PTEN mutant non-small-cell lung cancer requires ATM to suppress proapoptotic signaling and evade radiotherapy. Pharmacologic inhibition of ATM via KU-60019 and AZD1390 restores and even synergizes with ionizing radiation in PTEN-deficient human and murine NSCLC cells as well in ex vivo organotypic lung tumor slice cultures [53].

Together, these findings suggested that gene variants in DDR genes, especially in the ATM gene, detected in cfDNA are associated with survival and/or TTC in advanced PCa patients. However, the effect of these gene variants on different treatment regimens, such as PARP inhibitors or inhibitors of single DDR genes (e.g., ATM inhibitors), must be studied in future prospective studies.

## Figures and Tables

**Table 1:** Kaplan–Meier analysis results showing the association of ATM, NBN and DDR gene variants with prognosis.

Parameter	Kaplan–Meier Analysis					
	OS		DSS		TTC	
	Months	*p*	Months	*p*	Months	*p*
ATM_CPS yes vs. no	72.0 vs. 140.2	**0.022**	72.0 vs. 145.1	**0.010**	6.8 vs. 14.9	**0.034**
ATM_FI yes vs. no		ns		ns		ns
NBN_CPS yes vs. no		ns		ns		ns
NBN_FI yes vs. no		ns		ns	9.2 vs. 17.1	**0.042**
DDR_genes with CPS median >4 vs. ≤4		ns		ns	9.7 vs. 16.9	**0.038**
DDR_genes with FI median >7 vs. ≤7		ns		ns		ns
DDR_Sum_CPS	nc	**0.008**	nc	**0.009**		ns
DDR_Sum_FI	nc	**0.007**	nc	**0.008**		ns

Abbreviations: ns, not significant; nc, not calculated because no patient died in the reference category. Significant *p* values are presented in bold font.

**Table 2:** Univariate Cox’s regression analysis results showing the association of ATM, NBN and DDR gene variants with prognosis.

Parameter	Univariate Cox’s Regression Analysis					
	OS		DSS		TTC	
	RR (95% CI)	*p*	RR (95% CI)	*p*	RR (95% CI)	*p*
ATM_CPS yes vs. no	3.96 (1.11–14.19)	**0.034**	4.82 (1.28–18.19)	**0.020**	2.72 (0.99–7.45)	(0.052)
ATM_FI yes vs. no		ns		ns		ns
NBN_CPS yes vs. no		ns		ns		ns
NBN_FI yes vs. no		ns		ns	2.19 (0.97–4.98)	(0.059)
DDR_genes with CPS median >4 vs. ≤4		ns		ns	2.14 (0.99–4.64)	(0.054)
DDR_genes with FI median >7 vs. ≤7		ns		ns		ns
DDR_Sum_CPS		nc		nc		ns
DDR_Sum_FI		nc		nc		ns

Abbreviations: 95% CI, 95% confidence interval; ns, not significant; nc, not calculated because no patient died in the reference category. Significant *p* values are presented in bold font.

## Data Availability

All data are available in the manuscript and the Supplementary Materials. Detailed datasets used and analyzed during the present study are available from the corresponding author upon reasonable request.

## Supplementary Materials

The following supporting information can be downloaded at: https://www.mdpi.com/article/10.3390/cells11223618/s1: Table S1: Clinicopathological data, treatment and study data; Table S2: Overview of sequence variants; and Table S3: DDR genes analyzed with CPS/FI variants and association with prognosis.

## Abbreviations

ATM	ATM serine/threonine kinase ataxia telangiectasia mutated gene
BPH	benign prostate hyperplasia
cfDNA	cell-free DNA
CPS	clinical pathological significance
ctDNA	cell-free tumor DNA
DDR	DNA damage repair
DSS	disease-specific survival
FI	functional impact
NBN	nibrin
mCRPC	metastatic castration resistant prostate cancer
OS	overall survival
PCa	prostate cancer
PI3K	phosphatidylinositol 3-kinase
TC	treatment change
TTC	time to treatment change
