# Supplementary material for: Cell-Free DNA Sequencing Reveals Gene Variants in DNA Damage Repair Genes Associated with Prognosis of Prostate Cancer Patients
Source: Cells. 2022 Nov 15;11(22):3618. doi: 10.3390/cells11223618 (PMC9688453; doi:10.3390/cells11223618)
Supplement: Supplementary file 1 [file cells-11-03618-s001.zip › Suppl. files/Supplemental Table S1.pdf]

Supplemental Table S1

Cell-Free DNA Sequencing reveals Gene Variants in DNA Damage Repair Genes associated with Prognosis in Prostate Cancer Patients

Verena Lieb[1,2], Amer Abdulrahman[1,2], Katrin Weigelt[1,2], Siegfried Hauch[3], Michael Gombert[3], Juan Guzman[1,2], Laura Bellut[1,2], Peter Goebell[1,2], Robert Stöhr[2,4], Arndt Hartmann[2,4], Bernd Wullich[1,2], Helge Taubert[1,2,5,*], Sven Wach[1,2,5]

Suppl. Table S1. Clinicopathological data, treatment and study data

Parameter	n
Patients	34
Samples	39
Age, median (range) in years	67 (48-89)
Metastasis	
cM0	3
cM1	31
cfDNA, mean (range) in ng/ml	17.93 (2.37-206.25)
CTC-positive patients (samples)	9 (11)
AR-V7-CTC-positive patients (samples)	3 (4)
Survival time, median (range) in months	48 (6-182)
Overall survival	
Alive	24
Dead	10
Disease-specific survival	
Yes	25
No	9
Time from blood sampling to last contact, median (range)	10 (0-61)
Time from metastasis to last follow up, median (range)	31 (11-133)
Time to treatment change	9 (1-40)
Treatment change	
Yes	24
No	8
Not known	2
Pretreatment	
None	6
Depriv	10
AE	8
Tax	3
Multiple	7
Treatment	
AE	10
Tax	18
Chemotherapy/hormone	6
Pretreatment + treatment	

None + chemotherapy/hormone	6
Depriv + AE	5
Tax + AE	4
Mult + AE	2
Depriv + Tax	4
AE + Tax	8
Mult + Tax	5

Abbreviations: None, no pretreatment; Depriv, hormone deprivation; AE, antiandrogens abiraterone/enzalutamide; Tax, taxanes; Mult, multiple (both AE and Tax). Chemotherapy/hormone following none, AE following Depriv, AE following Tax, AE following Mult, Tax following Depriv, Tax following AE, Tax following Mult.
